# Expanding horizons in *Endocrine-Related Cancer*: innovation, imagination, and a shared global vision

**DOI:** 10.1530/ERC-25-0497

**Published:** 2026-01-02

**Authors:** Karel Pacak

**Affiliations:** ^1^ AKESO holding, Prague, Czech Republic; ^2^ Palacky University, Olomouc, Czech Republic

*‘Bringing something new begins when we change minds, not just add skills.’*

It is with deep honour and humility that I step into the role of the sixth Editor-in-Chief of *Endocrine-Related Cancer* (ERC). I am profoundly aware of the legacy I inherit from my distinguished predecessors, Professors Vivian James, Marc Lippman, James Fagin, Charis Eng, and most recently Matthew Ringel, each of whom shaped ERC into a flagship journal that embodies excellence, rigour, and vision. Their contributions have built a strong foundation, elevating ERC into a trusted forum where basic and clinical science meet, progress, and further evolve. It is on these strong shoulders that I now stand, committed to carrying forward the legacy of my predecessors while also imagining new horizons for our field.

Unlike more common cancers, many endocrine tumours are rare. They represent a unique and rapidly evolving field at the interface of endocrinology and oncology. Their rarity brings challenges, including small patient numbers, limited clinical trials, scarce evidence from single centres or countries, and often restricted funding. Yet these very challenges call us to think globally, to join efforts across institutions and continents, to accelerate discovery and innovation, and to build international registries. ERC is uniquely positioned to serve as a catalyst for this mission. Building on this global mission, we will promote advances in molecular biology, proteomics, biochemistry, and functional imaging that are transforming how we understand tumour pathogenesis and clinical behaviour. Increasingly, the immune system and its interplay with endocrine tumours offer other fresh insights and therapeutic opportunities. Artificial intelligence and machine learning now accelerate this progress, enabling rapid translation of discoveries into patient care for these tumours. Equally, we will foster the generation and dissemination of data from clinical trials, as well as the development of guidelines and consensus statements, to ensure that scientific progress is matched by meaningful improvements in patient care worldwide.

## A vision for ERC

As I begin this new chapter, I envision ERC not only as a journal but as a platform for collaboration, inspiration, and progress. To achieve this, I propose five guiding pillars:

### 1. Excellence and rigour

ERC’s reputation rests on its rigorous and fair peer review, high ethical standards, and commitment to robust science. We will preserve and strengthen these principles while exploring new models, such as double-anonymous peer review, which can further increase fairness and transparency. Every paper published in ERC should stand as a testament to robust and ethical science.

### 2. Inclusivity and diversity

Science thrives on diversity of thought, discipline, geography, and background. ERC will encourage the inclusion of young scientists and clinicians in its processes, provide opportunities for early-career investigators, and ensure that our Editorial Board reflects a truly international and multidisciplinary community.

### 3. Globalisation and collaboration

ERC recognises that meaningful progress in endocrine oncology relies on global cooperation. Through its pages, the journal will highlight and disseminate the outcomes of international registries, large multinational and global studies, and data-sharing initiatives, including those focusing on rare endocrine tumours. Such contributions will be welcomed, as they address geographical, genetic, environmental, healthcare-system, and socioeconomic differences that influence disease presentation, management, and outcomes across populations. By providing a platform for these collective achievements, ERC will promote visibility, shared learning, and dialogue that advance evidence-based guidelines and ultimately improve patient care worldwide.

### 4. Innovation and technology

Our field is entering an era shaped by advanced technologies. ERC will broaden its scope to highlight areas such as artificial intelligence and machine learning, nanotechnologies, novel molecular biology techniques, functional imaging, and immunotherapies. These approaches are redefining how we diagnose, classify, and treat endocrine cancers, and ERC must remain at the forefront of publishing them. New sections, such as ‘Clinical Review: Approach to Patient with Endocrine Tumour X’, ‘Hot Topics’, and ‘Endocrine Tumour in Focus’, will give space to fresh voices, innovative ideas, and diverse perspectives.

### 5. Imagination and vision

Scientific progress requires more than data; it requires vision. ERC will encourage bold thinking, creative hypotheses, and new perspectives that push boundaries. By providing space for pioneering ideas, we can inspire the next generation of endocrine cancer research. Our role is not only to document what has been achieved but to inspire what can be imagined. To capture and foster this spirit, the journal will introduce a new article series, ‘Advances in Endocrine Tumours’, dedicated to visionary ideas and forward-looking discussion.

## Looking ahead

To achieve this vision, ERC will expand its Editorial Board to include experts whose expertise bridges multiple disciplines: leaders in oncology-endocrinology, radiology and nuclear medicine, endocrine surgery, immunology, artificial intelligence and machine learning, and clinical trials. Many of these individuals bring dual or cross-speciality experience, reflecting the increasingly integrated nature of modern endocrine oncology. We will continue to support rapid publication timelines while also encouraging comprehensive, in-depth reviews. ERC will further strengthen its role as a global forum where scientists and clinicians come together not only to share discoveries but also to create new networks, guidelines, and collaborations that extend far beyond the pages of the journal.

## A collective mission

I am deeply grateful to the ERC Editorial Board, Bioscientifica, and the Society for Endocrinology for their trust and support. Most importantly, I call upon you, our readers, authors, and reviewers, to join in this mission. ERC can only flourish if it remains a community endeavour, rooted in rigorous science but guided by imagination, collaboration, and compassion for the patients whose lives depend on our work. Finally, we will continue to strengthen our partnerships with sister societies across the world, ensuring that ERC sustains its position as a leading and influential journal in the field.

As I embark on this journey, I am reminded of a guiding principle: *‘What we do for ourselves dies with us. What we do for others is immortal’.* May ERC continue to expand horizons, inspire discovery, and strengthen our global community, transforming imagination into innovation, and innovation into improved care for patients around the world. Together, let us build a future where science, creativity, and humanity come together for the benefit of patients worldwide.

## Declaration of interest

The author declares that there is no conflict of interest that could be perceived as prejudicing the impartiality of the work reported.

## Funding

This work did not receive any specific grant from any funding agency in the public, commercial, or not-for-profit sector.

